# Update and Application of a Deep Learning Model for the Prediction of Interactions between Drugs Used by Patients with Multiple Sclerosis

**DOI:** 10.3390/pharmaceutics16010003

**Published:** 2023-12-19

**Authors:** Michael Hecker, Niklas Frahm, Uwe Klaus Zettl

**Affiliations:** Division of Neuroimmunology, Department of Neurology, Rostock University Medical Center, Gehlsheimer Str. 20, 18147 Rostock, Germany; niklas.frahm@med.uni-rostock.de (N.F.); uwe.zettl@med.uni-rostock.de (U.K.Z.)

**Keywords:** deep learning, drug-drug interactions, drug-food interactions, drug safety, multiple sclerosis, prediction model, structural similarity

## Abstract

Patients with multiple sclerosis (MS) often take multiple drugs at the same time to modify the course of disease, alleviate neurological symptoms and manage co-existing conditions. A major consequence for a patient taking different medications is a higher risk of treatment failure and side effects. This is because a drug may alter the pharmacokinetic and/or pharmacodynamic properties of another drug, which is referred to as drug-drug interaction (DDI). We aimed to predict interactions of drugs that are used by patients with MS based on a deep neural network (DNN) using structural information as input. We further aimed to identify potential drug-food interactions (DFIs), which can affect drug efficacy and patient safety as well. We used DeepDDI, a multi-label classification model of specific DDI types, to predict changes in pharmacological effects and/or the risk of adverse drug events when two or more drugs are taken together. The original model with ~34 million trainable parameters was updated using >1 million DDIs recorded in the DrugBank database. Structure data of food components were obtained from the FooDB database. The medication plans of patients with MS (*n* = 627) were then searched for pairwise interactions between drug and food compounds. The updated DeepDDI model achieved accuracies of 92.2% and 92.1% on the validation and testing sets, respectively. The patients with MS used 312 different small molecule drugs as prescription or over-the-counter medications. In the medication plans, we identified 3748 DDIs in DrugBank and 13,365 DDIs using DeepDDI. At least one DDI was found for most patients (*n* = 509 or 81.2% based on the DNN model). The predictions revealed that many patients would be at increased risk of bleeding and bradycardic complications due to a potential DDI if they were to start a disease-modifying therapy with cladribine (*n* = 242 or 38.6%) and fingolimod (*n* = 279 or 44.5%), respectively. We also obtained numerous potential interactions for Bruton’s tyrosine kinase inhibitors that are in clinical development for MS, such as evobrutinib (*n* = 434 DDIs). Food sources most often related to DFIs were corn (*n* = 5456 DFIs) and cow’s milk (*n* = 4243 DFIs). We demonstrate that deep learning techniques can exploit chemical structure similarity to accurately predict DDIs and DFIs in patients with MS. Our study specifies drug pairs that potentially interact, suggests mechanisms causing adverse drug effects, informs about whether interacting drugs can be replaced with alternative drugs to avoid critical DDIs and provides dietary recommendations for MS patients who are taking certain drugs.

## 1. Background

Multiple sclerosis (MS) is a chronic immune-mediated neurodegenerative disease of the central nervous system (CNS), which affects over 2.8 million people worldwide [1,2]. The typical age of onset of MS is between 20 and 40 years [1]. Symptomatology and disease severity vary widely among individuals, making it challenging to predict long-term outcomes [1,3]. A single episode of neurological symptoms that is consistent with MS but does not fully meet the criteria for a diagnosis of MS is called clinically isolated syndrome (CIS) [4,5]. The most common disease course is relapsing-remitting MS (RRMS), which is characterized by unpredictable episodes of new or worsening symptoms followed by periods of recovery. About 85–90% of MS cases begin with this course [6]. The RRMS phase may later transition into secondary progressive MS (SPMS), where neurologic dysfunction accumulates without intermittent recovery. Approximately 10–15% of patients show a progressive worsening of disability in the absence of acute exacerbations from onset, which is referred to as primary progressive MS (PPMS) [1,4].

The treatment of MS requires a multifaceted approach and includes disease-modifying therapy, acute relapse treatment, symptomatic treatments, comorbidity management, rehabilitation interventions, psychological support and lifestyle modifications [7]. Disease-modifying drugs (DMDs) play a crucial role in reducing inflammatory disease activity, preventing relapses and slowing disease progression to maintain a better quality of life. More than a dozen DMDs with different mechanisms of action, routes of administration and degrees of efficacies have been approved for MS [8,9,10]. They primarily act by suppression or modulation of immune function [11]. However, undesirable side effects may occur during the course of treatment, which frequently prompts DMD therapy switches [7,12,13]. While the landscape of DMDs for MS has evolved tremendously over the past 30 years, intense efforts have also been made to identify novel drug targets. One promising target is the enzyme Bruton’s tyrosine kinase (BTK), which is centrally involved in the development and activation of B cells as well as myeloid cells [14]. Inhibition of BTK thus emerged as a therapeutic strategy for both relapsing and progressive forms of MS [15] and other diseases [14]. Several oral BTK inhibitors (BTKis) are currently being evaluated in clinical trials for MS with regard to their efficacy and safety [16,17]. However, in addition to disease-modifying therapies, a broad spectrum of drugs is available to treat the various symptoms of MS (e.g., spasticity, pain and loss of bladder control) and co-existing conditions (e.g., psychiatric disorders and hypertension) [3]. Self-medication with over-the-counter (OTC) drugs and complementary and alternative medicines (e.g., vitamin and mineral supplements) is also widely practiced [18,19]. The use of multiple drugs (i.e., polypharmacy) is therefore common. In fact, 15% to 59% of patients with MS take at least five drugs at the same time [20].

The concurrent use of multiple drugs carries the inherent risk of drug-drug interactions (DDIs). Using a database-driven approach, we have found a prevalence of potential DDIs of 70% in MS patients [21]. DDIs can lead to undesired drug exposure and adverse clinical outcomes. They are generally classified into pharmacokinetic and pharmacodynamic interactions. Pharmacokinetic interactions affect the liberation, absorption, distribution, metabolism or excretion of drugs. The inhibition or induction of drug-metabolizing enzymes is a major cause of such DDIs [22]. Pharmacodynamic interactions occur when the pharmacological effect of one drug is altered by that of another drug, resulting in enhanced or diminished therapeutic efficacy and/or side effects [23]. Drug responses can also be significantly altered when consuming certain foods or beverages, which is referred to as drug-food interactions (DFIs) [24]. The recognition of DDIs and DFIs is crucial in the treatment of patients to optimize therapeutic benefits, enhance safety and mitigate unnecessary healthcare costs. However, drug interactions are difficult to identify during clinical trials, which usually involve a limited number of participants with a restricted range of drug combinations and do not well represent the broader population with its diverse comorbidities and medication regimens [25]. On the other hand, experimental screens to detect potentially interacting drugs are demanding in terms of cost and time [26,27,28]. Advanced tools for evaluating the potential for DDIs and DFIs are thus essential for risk assessment, treatment optimization and drug development.

Several DDI databases and screening programs can be used as clinical decision support tools [29,30]. They mainly rely on information from drug labels and the scientific literature (e.g., case reports) and provide clinicians with annotations on the mechanisms and consequences of DDIs and DFIs. However, DDI resources are often commercial systems, and there is considerable discordance among them in the detection and classification of potential DDIs due to a lack of standards for DDI evidence evaluation [31]. In a comparison of three databases, we have found an overlap of detected potential DDIs of only 18.9% [21]. It is thus recommended to use more than one database [32,33], but knowledge of DDIs for new agents (such as BTKis) is generally incomplete and inconsistent. On the other hand, there is a disparity between potential and clinically relevant DDIs, and only a small proportion of DDIs result in actual patient harm [34,35]. To assist in DDI discovery and efficiently test large numbers of drug pairs, computational approaches have been developed [36,37,38] (Box 1). A recent review lists 46 deep and graph learning models for predicting DDIs (30 for binary classification and 16 for multi-class/multi-label classification) that have been published since 2018 [38]. These models are often based on structural and other similarities and the assumption that similar drugs have similar interaction patterns. For more detailed information, the reader is referred to comprehensive reviews [36,37,38].

Box 1 **Machine learning approaches for DDI prediction in a nutshell.**

First attempts to computationally infer novel potential DDIs based on data from electronic medical records, adverse event reports, scientific abstracts and other sources were made about 10 years ago [39,40]. In recent years, deep neural networks and graph neural networks have been actively developed for DDI prediction because of their superior performance compared to traditional machine learning techniques, especially due to their ability to automatically learn intricate features from unstructured data. These neural networks are often designed not only to identify drug pairs that may interact (binary classification) but also to make predictions about what the exact type of interaction or side effect may be (multi-class/multi-label classification) [37]. Pioneers of these types of models are DeepDDI [41] and Decagon [42], which achieved a mean accuracy of 92.4% and an area under the receiver operating characteristic curve of 0.872, respectively. Subsequently, more than 40 deep and graph learning models for DDI prediction, which differ in the input data (e.g., molecular structures or a DDI network), the featurization of input data (e.g., via dimensionality reduction or autoencoder embeddings) and the architecture of the neural network (e.g., multi-layer perceptron or graph attention network), have been published [37,38]. They can be broadly divided into chemical structure-based, network-based, natural language processing-based and hybrid models [38]. A main research direction is to improve DDI prediction performance by considering multiple data modalities that provide complementary information. An example is DDIMDL, which uses four types of drug features: chemical substructures, targets, enzymes and pathways [43]. The variety of methods that have been developed in a short period of time is overwhelming, and it is not straightforward to say which concept is the best. However, the accuracy of a DDI prediction model always strongly depends on the amount and quality of data on which it was trained. It is therefore remarkable that the volume of data that are available in drug databases has expanded considerably in the past few years [44]. Despite these advances, patient-specific factors (e.g., medical conditions) and pharmacologic factors (e.g., drug dosages) need to be better taken into account for a more practical use of DDI prediction models in identifying patients at particular risk of severe adverse drug effects.

A state-of-the-art deep learning framework and benchmark for DDI/DFI prediction methods is DeepDDI [41]. DeepDDI takes structural information and the names of drug and food compounds as inputs, calculates pairwise structural similarity scores based on molecular fingerprints, reduces the features’ dimensionality using principal component analysis (PCA) and feeds them to a deep neural network (DNN). The DNN consists of nine hidden layers, and each hidden layer has 2048 nodes with rectified linear unit activation function. The DNN generates human-readable sentences as outputs, providing specific descriptions of DDIs and DFIs in terms of changes in pharmacological effects and/or risk of adverse drug effects. DeepDDI was designed as a multi-label classification model to predict 86 different DDI types with a mean accuracy of 92.4% [41]. As an advantage, it does not require detailed drug information (e.g., side effects), which are often unavailable for drugs in clinical trials. However, its use is limited to small molecule compounds. Moreover, the model was trained on a DDI dataset from DrugBank version 5.0.3 that was released in October 2016 and since then, the number of DDIs in DrugBank has expanded considerably, forming a much denser DDI network [44,45].

In this study, we used for the first time a deep learning-based approach to specifically investigate DDIs and DFIs involving small molecule DMDs, BTKis and other drugs used by patients with MS. For this purpose, we have updated the DeepDDI model and analyzed the predictions to explore DDIs that may occur in patients after switching disease-modifying treatment and potential mechanisms causing adverse drug effects, as well as drug interactions with food compounds. Our study delivers useful information on which drugs might be replaced by alternative drugs and which foods might be avoided during specific medication. Such information can guide physicians in carefully reviewing undesired and preventable potential drug interactions, which is an important aspect given the complex and diverse treatment regimens often utilized in MS.

## 2. Methods

### 2.1. Patient Cohort

To capture the spectrum of drugs taken by MS patients and to assess the prevalence of predicted DDIs, we used the medication schedules from a patient cohort that we have recently described elsewhere [21,46,47]. The cohort consisted of 627 patients and was recruited at the Department of Neurology of the Rostock University Medical Center and at the Ecumenic Hainich Hospital Mühlhausen (Mühlhausen, Germany). The female-to-male ratio was 2.4, and the age of the patients ranged from 19 to 86 years. The study population included cases with CIS (*n* = 27), RRMS (*n* = 388), SPMS (*n* = 154) and PPMS (*n* = 58). The diagnosis of MS was confirmed according to the revised McDonald criteria [5]. The patients’ degree of disability, which was evaluated using the Expanded Disability Status Scale (EDSS) [48], averaged a score of 3.6. At a median disease duration of 10 years, 70.7% of the patients (*n* = 443) had at least one comorbidity in addition to MS (Table 1). Patient care and treatment followed routine clinical practice. For further details on the sociodemographic and clinical data of the patients, we refer to our previous publications [21,46,47].

Data on pharmacologic interventions were obtained from the medical records and structured patient interviews. For each patient, all medications were recorded with brand names and generic names of the active ingredients, independent of the treatment goals. We thus considered long-term medications, on-demand medications and acute medications that were used in the treatment of MS or other conditions. We also considered both prescription (Rx) as well as non-prescription (OTC) drugs and dietary supplements [21,46,47].

Written informed consent was obtained from all patients prior to data collection. The study was performed with approval from the ethics committees of the University of Rostock and of the State Medical Association of Thuringia (approval numbers A 2014-0089 and A 2019-0048) and in accordance with the Declaration of Helsinki.

### 2.2. Drug Data and Gold Standard DDI Dataset

For all drugs taken by the patients, the corresponding entries were searched in the DrugBank database version 5.1.10 (released in January 2023) [45]. In the case of combination drugs, the contained active ingredients were searched (e.g., ethinylestradiol and levonorgestrel as components of combined oral contraceptives). Additionally, we considered all DMDs approved for the treatment of MS by the US Food and Drug Administration and/or the European Medicines Agency as of June 2023 [8,10,49,50] (Table 2). Moreover, as BTKis may provide attractive therapeutic benefits in MS by targeting both innate and adaptive immune responses, although their long-term efficacy and potential side effects remain to be explored, we also included all BTKis under investigation for the treatment of MS in phase 2 or phase 3 clinical trials [15,16,17] (Table 2). For the small molecule DMDs and the selected BTKis, the chemical structures according to DrugBank [45] or PubChem [51] have been visualized in Supplemental Figure S1.

DrugBank is a comprehensive resource that provides detailed information about thousands of drugs, such as chemical properties, drug targets, pharmacological actions and manually sourced DDIs [45]. With permission, we downloaded the complete database to compile an up-to-date gold standard DDI dataset for further training of the DeepDDI model. DeepDDI distinguishes 86 important DDI types [41], whose general sentence structures are given in Supplementary Table S1. We have extracted from DrugBank all DDIs that belong to these DDI types and where both drugs of a pair have an appropriate representation of the molecular structure in the simplified molecular-input line-entry system (SMILES). Unlike in earlier DrugBank releases, only one DDI type is specified for each drug pair, which should be noted with regard to the new gold standard.

### 2.3. Food Content Data

Information on food contents was obtained from FooDB (pre-release 1.0 from April 2020), the largest public database on the composition of foods and beverages that contains a vast collection of food compounds, including macronutrients, micronutrients and phytochemicals (https://foodb.ca, accessed on 5 December 2022). For the prediction of DFIs, we extracted all food sources with quantitative content data and the associated food compounds with their names and chemical structures.

### 2.4. DeepDDI Model Update and Performance Evaluation

**Figure 1:**
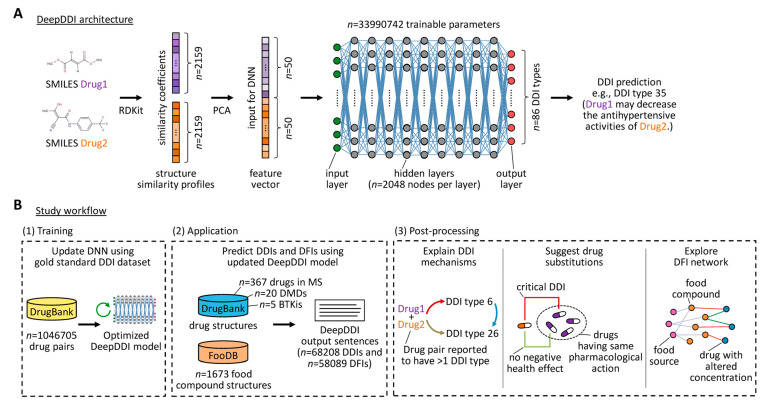
Deep learning framework for the prediction of drug interactions and study overview. (**A**) For the computational analysis of drug interactions that may occur in patients with multiple sclerosis, we used the DeepDDI method developed by Ryu et al. [41]. DeepDDI takes the names of drug-drug or drug-food compound pairs and their molecular structures in SMILES notation as input and predicts 86 different DDI types in the form of human-readable sentences as output. The chemical structures are processed to Morgan fingerprints and then compared with 2159 drug structures by calculating Dice similarity coefficients. The dimension of the structural similarity profiles is then reduced to 50 using PCA, yielding a combined feature vector of size 100 as input for the DNN. DeepDDI is a multi-label classification model that can predict multiple DDI types at the same time for a given compound pair of interest. (**B**) We have trained the original model for another 20 epochs using >1 million DDIs as the new gold standard based on DrugBank release 5.1.10. This improved the prediction accuracy in the testing set to 92.14%. The updated model was then applied to systematically predict DDIs and DFIs for drugs used by patients with MS and drugs approved or under investigation for the treatment of MS, given that their structure is available in SMILES. Information on 1673 food compounds with associated food sources was obtained from FooDB pre-release 1.0. The results were further analyzed in 3 post-processing steps to predict DDI mechanisms causing adverse effects, to suggest alternative drugs for avoiding negative health effects and to explore food compounds that potentially interfere with the pharmacological action of drugs. BTKi = Bruton’s tyrosine kinase inhibitor under investigation in MS, DDI = drug-drug interaction, DFI = drug-food interaction, DMD = disease-modifying drug approved for the treatment of MS, DNN = deep neural network, MS = multiple sclerosis, PCA = principal component analysis, SMILES = simplified molecular-input line-entry system.

We have chosen DeepDDI because it is (1) a multi-label classification model that (2) does not require detailed drug information (3) for predicting both DDIs and DFIs with (4) reasonable accuracy (see Background section) and because of (5) the accessibility of the source code and model (https://bitbucket.org/kaistsystemsbiology/deepddi, accessed on 1 December 2022). The DNN of DeepDDI was originally trained with 192,284 DDIs from DrugBank release 5.0.3 [41,45]. Since then, the number of known DDIs has grown considerably. An update of the model was therefore necessary. For this purpose, a structure similarity profile was calculated for each drug in the new gold standard using 2159 drugs as a fixed comparison target as described in the original paper [41]. In this step, the structural similarity between each pair of molecules was evaluated by computing Dice coefficients on the basis of their circular fingerprints, which were generated using the Morgan algorithm with radius 2 [52]. This was done with the RDKit version 2021.9.4 for Python. The dimensionality of the structure similarity profiles was then reduced to 50 by principal component analysis. Consequently, for each drug pair, a combined feature vector of length 100 resulted as input for the DNN (Figure 1). The feature vectors of the DrugBank-contained DDIs were then used for updating the pre-trained DeepDDI model. In doing so, we essentially applied the hyperparameters defined by Ryu et al. [41]. Accordingly, the gold standard dataset was randomly split into a training set (60%), a validation set (20%) and a testing set (20%). The ~34 million trainable parameters of the DNN were adapted to minimize the prediction error using binary cross-entropy as loss function and the Adam algorithm for optimization [53]. Batch normalization was used, and the learning rate was set to 0.001. However, we chose a larger batch size of 1024 and a smaller number of epochs of 20 than in the initial training. The update was performed in the Python 3.7 environment with the Keras interface for the TensorFlow library (version 2.6.4) [54] on a P100 graphics processing unit via the Kaggle platform (https://kaggle.com, accessed on 1 February 2023). For performance evaluation, we finally determined loss and categorical accuracy for the training, validation and testing sets.

### 2.5. Prediction of DDIs and DFIs

The updated model was then applied to predict DDIs and DFIs for all possible drug-drug and drug-food compound pairs using as input their chemical structures provided in canonical SMILES (Figure 1). In addition to the small molecule drugs used by the MS patients, DMDs and BTKis, further drugs were included to be able to later suggest alternative drugs with a lower likelihood of causing critical DDIs. Critical DDIs are those that were previously regarded as having clear negative health effects [41], namely, the 14 DDI types 18–23 and 25–32 (Supplemental Table S1). In order to be considered as an alternative candidate for another drug, an approved drug was required to have the same known pharmacological action(s) (e.g., agonist or inhibitor) with the same protein target(s) reported in DrugBank. For DFIs, the same 86 types were considered as for DDIs, since they were predicted with the same DNN model. However, an additional validation step was carried out as implemented by Ryu et al. [41]: A predicted DFI was only considered if there was an approved drug that was structurally similar to the food component with a Dice coefficient of ≥0.75 and for which there was an interaction of the same type in the DrugBank dataset. DFI output sentences with the food compound as the subject indicate that it may affect the pharmacological effects of the interacting drug. Otherwise, they indicate a potential bioactivity of the food compound that is modulated by the drug. Unlike in the original implementation, we have doubled the threshold for output neuron activity to obtain final predictions for DDIs and DFIs.

### 2.6. Post-Processing of DDI Results

The high-confidence DDI/DFI predictions were further processed in R version 4.1.2. Firstly, we determined the percentage of MS patients that had a predicted DDI in their medication plans. Secondly, we examined with how many non-DMDs the patients were taking, the DMDs and BTKis that are approved and investigated for the therapy of MS, respectively, would potentially provoke a DDI of a particular type. Thirdly, we assessed the proportion of patients who would be at risk of a particular DDI type if they were to stop their current DMD therapy and switch to another DMD or a BTKi (or start such a therapy), given all the other medications they were taking. The resulting DDI frequencies were visualized in bar charts and as a heat map.

We have also utilized the output sentences for the prediction of potential causal mechanisms of unintended pharmacological effects (Figure 1). Most DDIs in DrugBank belong to DDI type 26, which indicates an increased risk or severity of adverse drug effects, with the underlying mechanism being unknown. A feature of DeepDDI is that it can predict more than one DDI type for a given drug combination [41]. We have therefore searched for pairs of drugs that were (1) taken together by some MS patients, for which (2) a DDI of type 26 was specified in DrugBank and predicted by DeepDDI and for which (3) another DDI of a different type was also predicted by the model. The latter DDI type may then explain the DDI of type 26.

Furthermore, we analyzed whether critical DDIs can be prevented by replacing a drug with an alternative drug having the same intended pharmacological effects, which means that the drugs presumably have similar mechanisms of action because they interact with the same target(s) (see previous section). For the new drug pair involving the alternative drug, it was required that the activation level of the DNN’s output neurons for all critical DDIs be <0.47, which is the default threshold [41]. On this basis, we derived recommendations to minimize negative health effects while maintaining the beneficial effects of each drug class.

### 2.7. DFI Network Visualization

The network analysis focused on DFIs potentially leading to changes in the in vivo concentrations of the small molecule drugs under scrutiny. For this purpose, we filtered output sentences describing a decrease or increase in the absorption (DFI types 1 or 2), bioavailability (DFI types 4 or 5), metabolism (DFI types 6 or 7) or serum concentration (DFI types 9 or 10) of a drug when combined with a food compound. We also identified up to 10 foods that are particularly rich in the respective food compounds according to FooDB. The relationships between the food sources, food components and drugs were then visualized using Cytoscape version 3.10.0 [55]. We finally highlighted food compounds predicted to commonly interact with either sphingosine-1-phosphate (S1P) receptor modulators for the therapy of MS or BTKis in clinical development for MS.

## 3. Results

### 3.1. Drugs Used by the Patients with MS

The patients (*n* = 627) were taking between 0 and 19 medications simultaneously. Most of the patients (*n* = 568, 90.6%) took at least two drugs and were thus at risk of a potential DDI. OTC products were used by 386 of the patients (61.6%). The proportion of patients receiving a DMD for MS was 62.0% (Table 1), and it was highest in patients with RRMS with 79.9% (CIS: 55.6%, SPMS: 35.7%, PPMS: 15.5%). After separating combination drugs into their active ingredients and assigning the drugs to DrugBank entries, a total of 367 different drug compounds were identified as being used by the patients (Supplemental Table S2). A subset of 312 of the drugs were small molecule drugs with well-documented chemical structure (*n* = 306 non-DMDs and *n* = 6 DMDs).

### 3.2. Update and Application of the DeepDDI Model

The DrugBank release 5.1.10 contains a total of 1,433,261 DDIs [45]. For 1,046,705 of these DDIs (73.0%), both drugs of the pair have an appropriate description of the molecular structure in SMILES and the DDI type is one of those distinguished by DeepDDI (Supplemental Table S1). These DDIs thus formed the new gold standard for the further training of the DNN in the present study. During model fitting, the loss decreased while the accuracy increased for the training and validation sets (*n* = 628,023 and *n* = 209,341 DDIs, respectively) as expected (Supplemental Figure S2). The updated model achieved a loss of 0.0071 and a categorical accuracy of 92.14% on the testing set (*n* = 209,341 DDIs). In a subsequent normal run of the DeepDDI program, 1,009,050 DDIs from the gold standard dataset (96.40%) were correctly predicted (Supplemental Figure S3).

The updated model was then applied to predict DDIs and DFIs. The analysis of DDIs focused on 322 small molecule drugs, namely, 306 non-DMDs used by the patients with MS (Supplemental Table S2) as well as 11 approved DMDs and 5 investigational BTKis (Table 2). For this number of drugs, 51,681 pairwise drug combinations are theoretically possible, and for 45,464 of them (88.0%), a DDI was predicted by DeepDDI. Two or more DDI types were predicted for 22,712 drug pairs, which resulted in a total of 68,208 DDIs (Supplemental Table S3). A subset of 15711 of these DDIs (23.0%) are included in DrugBank (i.e., known), while 52,497 DDIs (77.0%) were newly predicted by DeepDDI (i.e., unknown). It is important to consider that not all DDIs are necessarily undesirable. For instance, seven of the patients used the combination of ramipril and torasemide for the management of hypertension. For this drug pair, DeepDDI predicted DDI type 67 (“Ramipril may increase the hypotensive activities of Torasemide.”) in line with the information provided by DrugBank [45]. It should also be noted that no DDI was recorded in DrugBank for the selected BTKis (Table 2). However, the DeepDDI output sentences revealed numerous potential interactions for evobrutinib (*n* = 434 DDIs), fenebrutinib (*n* = 425 DDIs), orelabrutinib (*n* = 426 DDIs), remibrutinib (*n* = 372 DDIs) and tolebrutinib (*n* = 379 DDIs). Using the drugs and the 1673 food compounds as input, we obtained 58,089 DFIs (Supplemental Table S4). These DFIs were associated with 326 food compounds. The food sources most often related to DFIs were corn (*n* = 5456 DFIs), cow’s milk (*n* = 4243 DFIs) and red bell pepper (*n* = 3986 DFIs).

### 3.3. DDIs of Potential Relevance in the Patients

**Figure 2:**
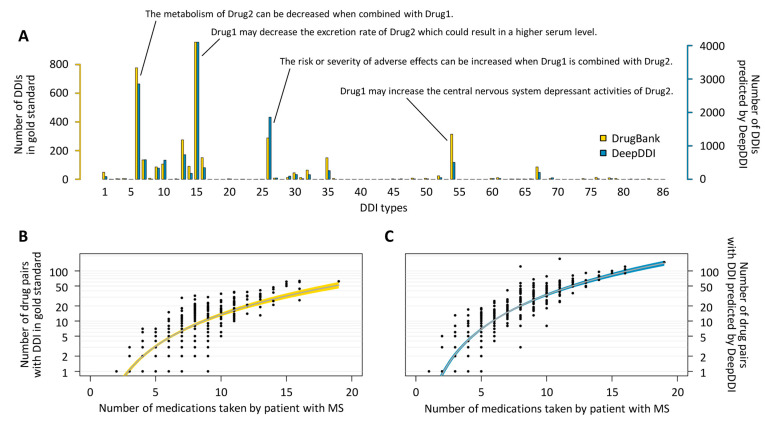
Evaluation of potential drug-drug interactions in patients with multiple sclerosis. The medication plans of patients with MS (*n* = 627) were searched for pairwise interactions between drug compounds with well-defined chemical structure. In total, we identified 3748 DDIs using the gold standard according to DrugBank release 5.1.10 and 13,365 DDIs using DeepDDI. For a given drug pair, exactly one DDI type is recorded in DrugBank, whereas DeepDDI may predict multiple DDI types at the same time. (**A**) Distribution of the frequency of DDI types in the gold standard and in the DeepDDI predictions. The output sentences for common DDI types are provided in the figure. (**B**) Number of potential DDIs in the gold standard and (**C**) according to DeepDDI results in relation to the number of medications taken. At least one potential DDI was found for most patients (*n* = 413 or 65.9% based on the gold standard and *n* = 509 or 81.2% based on the DNN model). For patients who used 10 or more medications (*n* = 73, 11.6%), we found at least 5 DDIs in either dataset. The increase in the number of DDIs with the extent of medication use is exponential. Fitted curves with 95% bootstrap confidence intervals are shown in yellow and blue, respectively. DDI = drug-drug interaction, DNN = deep neural network, MS = multiple sclerosis.

For the entire patient cohort, the number of possible pairwise combinations of coadministered small molecule drugs was 10,289 when counting with repetitions. A DDI was identified in the gold standard and in the DeepDDI output for a subset of 3748 and 8832 of these combinations, respectively. Overall, the DNN model yielded 13,365 DDIs, as two DDI types were predicted for 4533 drug combinations. The most abundant DDI types were type 15 (*n* = 4103 DDIs), type 6 (*n* = 2854 DDIs) and type 26 (*n* = 1858 DDIs) (Figure 2A, Supplemental Table S3). Drugs that were used exclusively for self-medication were involved in 1281 of the predicted DDIs (9.6%). Examples of such DDIs with OTC drugs and dietary supplements are “The metabolism of Dalfampridine can be decreased when combined with Curcumin.” (DDI type 6), “Sodium bicarbonate may decrease the excretion rate of Pantoprazole which could result in a higher serum level.” (DDI type 15) and “Zinc may increase the hypotensive activities of Doxazosin.” (DDI type 67).

Based on the gold standard from DrugBank, at least one DDI was found for 413 patients (65.9%). In comparison, according to the DeepDDI predictions, the DDI prevalence was as high as 81.2% (*n* = 509 patients), and it was particularly high in patients with progressive MS (CIS: 81.5%, RRMS: 73.2%, SPMS: 94.8%, PPMS: 98.3%). The latter reflects the higher medication use in these patients (CIS: 3.7 ± 2.1, RRMS: 4.1 ± 2.6, SPMS: 7.7 ± 3.5, PPMS: 7.6 ± 3.1 medications on average). Indeed, the number of potential DDIs exponentially increases with the number of medications taken (Figure 2B,C). Patients on ≥5 drugs (*n* = 334 or 53.3%) had at least one predicted DDI.

**Figure 3:**
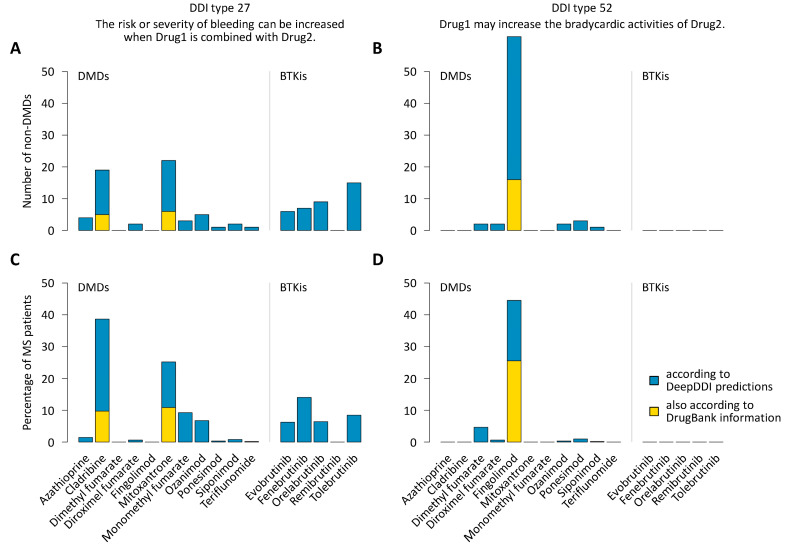
Prevalence of selected types of drug-drug interactions. The patients with MS (*n* = 627) used 306 different small molecule drugs that were not DMDs. (**A**,**B**) It is shown how many of the 306 non-DMDs are expected to cause a DDI of type 27 or type 52 when used in combination with an approved or investigational small molecule therapeutic agent for MS. (**C**,**D**) The bar charts depict the proportion of patients who would be at additional risk of DDI type 27 or DDI type 52 when receiving a particular DMD or BTKi after discontinuing the current DMD therapy. The predictions using the updated DeepDDI model (blue bars) revealed that many patients would be at increased risk of bleeding and bradycardic complications due to a potential DDI when taking cladribine (*n* = 242 or 38.6%) and fingolimod (*n* = 279 or 44.5%), respectively. DDIs of type 27 were also predicted for BTKis. The yellow bars indicate the subset of predicted DDIs that are also listed in the DrugBank database. BTKi = Bruton’s tyrosine kinase inhibitor under investigation in MS, DDI = drug-drug interaction, DMD = disease-modifying drug approved for the treatment of MS, MS = multiple sclerosis.

When all conceivable drug pairs were considered, regardless of whether or not the drugs were used together by any patient, numerous DDIs were predicted between DMDs for the treatment of MS and the 306 small molecule non-DMDs the patients were taking (Supplemental Table S3). This was equally true for the BTKis. Notably, DDIs involving BTKis were less often related to drug excretion (DDI types 15 and 16) but more often related to drug metabolism (DDI types 6 and 7) as compared with DDIs involving DMDs. Moreover, the BTKis were significantly less frequently associated with an increased risk or severity of adverse effects (DDI type 26) and an increase in CNS depressant drug activities (DDI type 54). There were no DDI predictions indicating increased hepatotoxic activities (DDI type 20) for any BTKi (Supplemental Figure S4). A closer look at individual DMDs revealed, for example, that cladribine and mitoxantrone were occasionally associated with an increased risk or severity of bleeding (DDI type 27, with *n* = 19 and *n* = 22 drugs, respectively), while fingolimod was frequently associated with an increase in bradycardic drug activities (DDI type 52, with *n* = 61 drugs) (Figure 3A,B). This was also reflected in the analysis in which we examined how many patients would have one or more additional DDI(s) if they were to switch to or start a therapy with a particular DMD or BTKi: We found that 38.6% and 44.5% of the patients would be at risk of a DDI of type 27 and type 52 when initiating the administration of cladribine and fingolimod, respectively (Figure 3C,D).

### 3.4. Mechanisms That May Explain Adverse Drug Effects

**Figure 4:**
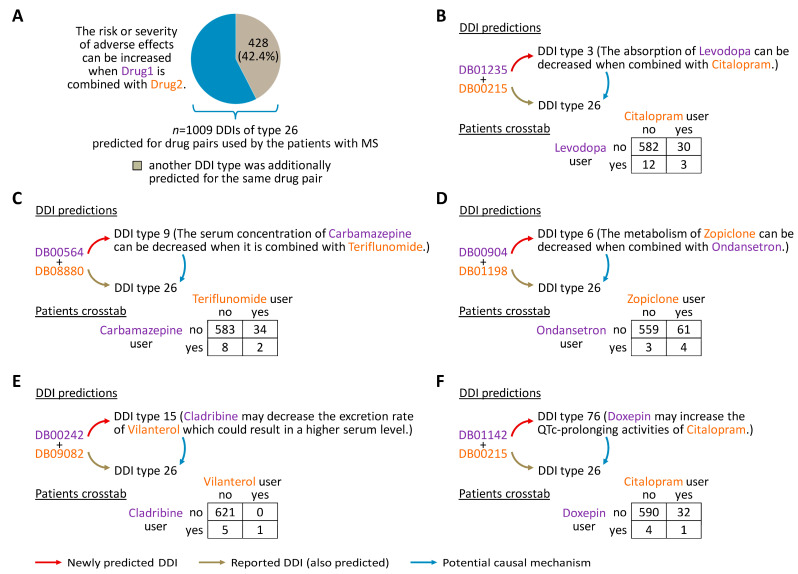
Prediction of potential causal mechanisms for adverse drug effects. (**A**) DeepDDI predicted a total of 1009 different DDIs of type 26 for pairwise drug combinations that were detected in the patient cohort (*n* = 627). For 428 drug pairs, another type of DDI was predicted at the same time by the multi-label classification model. (**B**–**F**) Examples of DNN output sentences that may explain DrugBank-contained DDIs of type 26 by pharmacokinetic or pharmacodynamic mechanisms. More specifically, it is suggested that the absorption, distribution, metabolism or excretion of some drugs may be altered by concomitantly administered drugs. As such DDIs may result in decreased efficacy or increased toxicity, they warrant the attention of healthcare professionals. For each drug compound pair, the contingency tables show the number of patients who used either drug or both. DDI = drug-drug interaction, DNN = deep neural network, MS = multiple sclerosis.

For drugs taken concurrently by the patients with MS, a total of 1009 different DDIs were predicted that had the expression “The risk or severity of adverse effects can be increased when Drug1 is combined with Drug2.” (DDI type 26). For 428 of these DDIs (42.4%), DeepDDI also assigned another DDI type for the same drug pair (Figure 4A, Supplemental Table S3). In this case, the output sentences may suggest DDI mechanisms that cause adverse effects by pointing to specific pharmacological effects. For instance, three patients were using levodopa together with citalopram. In addition to predicting DDI type 26 for this drug combination, in agreement with the information provided by DrugBank [45], the model generated the output sentence “The absorption of Levodopa can be decreased when combined with Citalopram.” (DDI type 3) (Figure 4B). Given this, an impaired absorption of levodopa in the intestine may lead to unpredictable response fluctuations, necessitating higher dosing with more pronounced adverse reactions. Another example is the prediction of “The metabolism of Zopiclone can be decreased when combined with Ondansetron.” (DDI type 6), which may explain the DDI of type 26 contained in DrugBank and also correctly predicted by DeepDDI for the same drug pair (Figure 4D). Here, both drugs may compete for the same cytochrome P450 (CYP) enzymes as substrates, resulting in higher drug concentrations, prolonged effects and an increased risk of drug toxicity. Further examples for the prediction of causal mechanisms potentially contributing to adverse drug events are shown in Figure 4.

### 3.5. Suggestions for Drug Substitution to Avoid Critical DDIs

**Figure 5:**
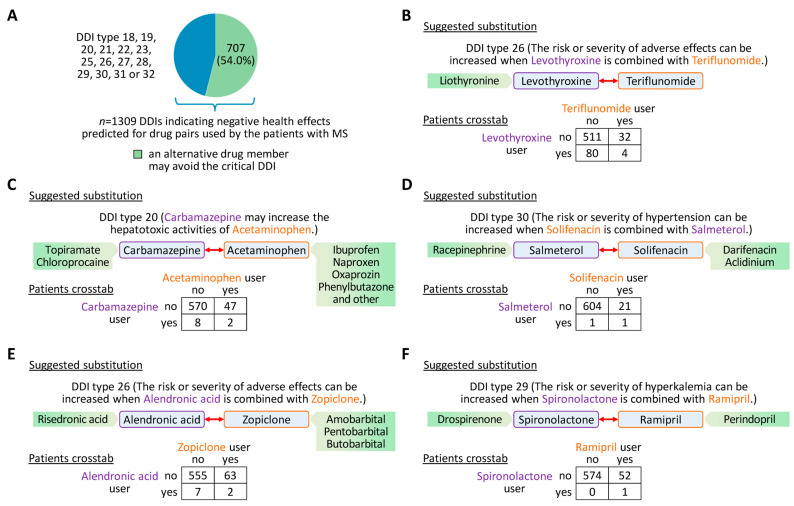
Suggestions for therapeutic substitution to avoid critical DDIs. (**A**) Based on the medication records of the patients (*n* = 627), DeepDDI predicted a total of 1309 DDIs corresponding to 14 DDI types associated with negative health effects. For 707 of those DDIs, alternative drugs with similar pharmacological effects could be suggested so that none of the critical DDI types is predicted for the candidate drug pair. (**B**–**F**) Examples of critical DDIs involving a DMD for MS (teriflunomide) or drugs for the treatment of MS-related symptoms (carbamazepine and solifenacin) or comorbid conditions. The green boxes contain drug members that were predicted to have a lower chance of such adverse effects, while presumably achieving the intended therapeutic effect. (**B**) For example, four patients were simultaneously taking teriflunomide and levothyroxine for which an increased risk or severity of adverse effects was predicted when used in combination. In this case, levothyroxine might be replaced by liothyronine, as this drug was not predicted to have a critical DDI with teriflunomide. However, the results should be interpreted with caution. It is important to note that alternative drugs were derived using only DrugBank information on the direct targets of drug action. Differences in drug potency, dosage, route of administration and indication require that the treating physicians must judge whether a change in therapy is really appropriate or not. For each drug compound pair, the contingency tables show the number of patients who used either drug or both. DDI = drug-drug interaction, DMD = disease-modifying drug, MS = multiple sclerosis.

For the patient cohort, a total of 1309 DDIs were predicted, which belong to the 14 DDI types associated with negative health effects (Supplemental Tables S1 and S3). In many cases (*n* = 707 or 54.0%), such DDIs were found to be preventable. In these cases, the replacement of a drug by another approved drug member having the same intended pharmacological effects led to different predictions according to which a critical DDI is unlikely to occur (Figure 5A). An example is the DDI “Carbamazepine may increase the hepatotoxic activities of Acetaminophen.” (DDI type 20), which was predicted for two of our patients (Figure 5C). When using as input for the DNN, e.g., topiramate as an alternative sodium channel blocker instead of carbamazepine or ibuprofen for pain relief instead of acetaminophen, the new candidate drug pair was not predicted to be associated with hepatic abnormalities or any other critical DDI type. As another example, the DDI “The risk or severity of hyperkalemia can be increased when Spironolactone is combined with Ramipril.” (DDI type 29) was predicted for one female patient (Figure 5F). However, the DeepDDI output suggests that drospirenone can substitute spironolactone and perindopril can be considered as an alternative for ramipril when treating high blood pressure. Either of these medication changes may reduce the risk of negative health effects while still acting on the same therapeutic targets. Further examples for the suggestion of alternative drugs are presented in Figure 5.

Between the BTKis in clinical development for MS and the non-DMDs, 416 critical DDIs were predicted, of which 112 (26.9%) possibly can be avoided by replacing the non-DMD (Supplemental Table S3). For instance, the risk or severity of bleeding was found to be increased when fenebrutinib is used in combination with acetylsalicylic acid and when tolebrutinib is used in combination with rivaroxaban (DDI type 27). To lower the chance of such a negative health effect, the results suggest that, e.g., acetaminophen or ibuprofen could be used instead of acetylsalicylic acid to reduce pain and fever, whereas bemiparin could be used as an antithrombotic agent instead of rivaroxaban when starting a treatment with the respective BTKis. Likewise, as an increased risk or severity of QTc prolongation was predicted for dimenhydrinate when taken together with evobrutinib (DDI type 32), it may be substituted, e.g., by meclizine, for which this DDI was not predicted.

### 3.6. DFIs That May Alter Drug Concentrations

**Figure 6:**
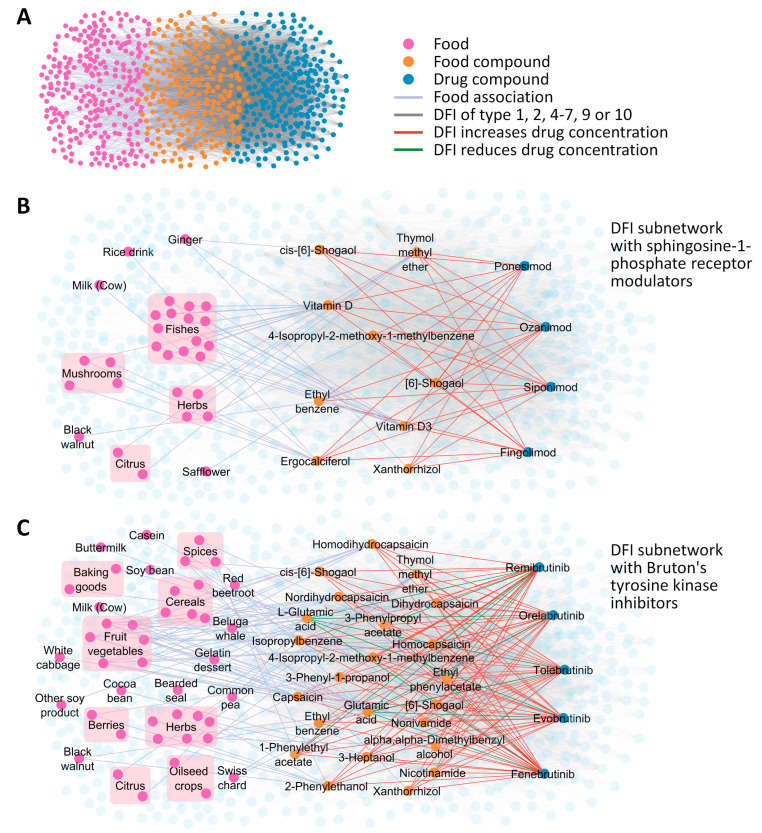
Network of drug-food interactions that may lead to changes in drug concentrations. Using the updated DeepDDI model, we obtained 58,089 DFIs for the small molecule drugs used by the patients with MS as well as the DMDs and BTKis included in this analysis. (**A**) This network shows a subset of 6860 DFIs that potentially result in increased or reduced in vivo concentrations of the drugs, i.e., their absorption, bioavailability, metabolism or serum concentration was predicted to be affected by food compounds. A wide variety of foods (*n* = 263) were identified among the top 10 sources of those food compounds (*n* = 227). (**B**) The same network in 2x magnification and highlighting DFIs with food compounds that commonly interact with S1P receptor modulators. In particular, the consumption of fish and mushrooms (grouped for better readability) was associated with increased concentrations of fingolimod, siponimod, ozanimod and ponesimod via vitamin D. (**C**) Subnetwork highlighting DFIs linked to BTKis. Most of these DFIs (*n* = 105 or 88.2%) putatively result in higher BTKi levels. However, glutamic acid, which is abundant in cereals, was consistently predicted to be associated with reduced levels of the BTKis. BTKi = Bruton’s tyrosine kinase inhibitor under investigation in MS, DFI = drug-food interaction, DMD = disease-modifying drug approved for the treatment of MS, MS = multiple sclerosis, S1P = sphingosine-1-phosphate.

In 6860 DFI output sentences, the food compound appeared as the subject and was predicted to reduce (*n* = 322 DFIs of type 1, 4, 7 or 9) or increase (*n* = 6538 DFIs of type 2, 5, 6 or 10) the in vivo concentration of a drug (Supplemental Table S4). Those DFIs connect 295 small molecule drugs (*n* = 280 non-DMDs used by the patients with MS, *n* = 10 DMDs and *n* = 5 BTKis) with 227 food compounds, for which we identified a total of 263 foods among the top 10 sources (Figure 6A). Exemplarily, a local network highlighting nine food compounds that were uniformly predicted to increase the level of S1P receptor modulators is visualized in Figure 6B. These food compounds included vitamin D from mushrooms and fishes (such as salmon and trout) and ethyl benzene from walnuts. For the BTKis, we found that their concentration can potentially be influenced jointly by 23 food compounds (Figure 6C). For instance, glutamic acid from cereals (such as wheat, oat and barley) and thymol methyl ether from citrus (such as lime and clementine) were associated through DFIs with reduced and increased BTKi levels, respectively. Such relationships indicate that certain foods may change the risk–benefit profile of the drugs when taken at the same time.

## 4. Discussion

The occurrence of DDIs and DFIs affects the treatment of many patients and has major implications not only for drug safety and therapy management, but also for the development of new drugs. However, there is a lack of systematic studies on DDIs/DFIs in patients with MS, who often receive complex treatment regimens already at a relatively young age. We have previously utilized drug interaction databases to identify potential DDIs in the medication plans of MS patients [21,46,47,56]. Here, we have applied for the first time a computational approach to effectively predict DDIs and DFIs specifically in the context of MS treatment. The multi-label deep learning model DeepDDI from Ryu et al. [41] was updated to accurately detect known DDIs and predict unknown DDIs/DFIs of various different types based on the structural similarity between drugs. We demonstrated that the predictions can provide important additional information for making treatment decisions in MS, which may contribute to minimizing adverse effects and maximizing therapeutic benefits in individual patients.

Using the updated DNN model, we found a DDI prevalence of 81.2% in the patient cohort (*n* = 627). This compares to up to 70.3% when using the database-driven approach [21,46,47]. Patients with progressive MS were at higher risk of potential DDIs than patients with CIS or RRMS due to the higher average number of medications taken. An older age, a higher number of comorbidities and a lower educational level are also strong predictors of having a DDI, as we have previously shown [21,46]. A key feature of the prediction model used in the present study is that it distinguishes between 86 DDI types. Such detailed reporting enables healthcare professionals to better educate patients on DDIs, to proactively monitor for changes in pharmacological effects and to adjust treatment plans if necessary. It has to be emphasized, however, that known and predicted DDIs should generally be regarded as early warnings because, even if valid, only a small number of them will be clinically relevant [29,34,35]. This is because multiple factors influence their practical significance. For instance, some DDIs may manifest only at high drug doses [57] or in a subset of patients who carry genetic variants that affect the function of drug-metabolizing enzymes [58]. Here, we did not follow up the patients for the actual occurrence of DDIs in terms of altered therapeutic efficacy or toxicity. We also did not analyze whether the treating physicians were aware of the DDIs and whether they deemed them beneficial (in case of synergistic interactions) or acceptable (in case of detrimental interactions) after considering patient-specific factors and carefully weighing the risks and benefits. It is possible that precautions had already been taken to reduce the risk of DDI-related adverse outcomes (e.g., by adjusting the dose or by ensuring an appropriate time interval between each two drug administrations). Further research is needed to evaluate how information on potential DDIs may influence treatment decisions and to determine the incidence of DDIs with clinical consequences in patients with MS.

We found various DDI types for the small molecule DMDs for MS in combination with the other Rx or OTC medications used by the patients. For example, DDIs of type 27, indicating an increased risk or severity of bleeding, were predicted for DMDs. Such DDIs can result from the concomitant use of drugs that interfere with blood clotting, e.g., by affecting the number or function of platelets. For mitoxantrone, a cytostatic agent that is still used in exceptional MS cases in Germany [10,59], reductions in platelet count were described after repeated infusions [60]. This appears to be compatible with DDIs of type 27 predicted for patients receiving mitoxantrone together with antiplatelet or anticoagulant drugs such as acetylsalicylic acid (*n* = 5) or rivaroxaban (*n* = 1) [61]. Cladribine administered orally in MS exhibits only minor effects on platelet counts [62], but in leukemia patients treated with intravenous or subcutaneous cladribine, transient thrombocytopenia is a common adverse event [63,64], rendering DDIs of type 27 more likely. This illustrates that the risk and severity of DDIs is influenced, e.g., by drug dosage, route of administration, treatment phase and the patients’ underlying conditions. Improved predictions might be achieved in the future by compiling a more specific gold standard DDI dataset and by filtering potential DDIs by drug indications and other aspects. However, this is challenging because, apart from the fact that there is for an active ingredient like cladribine only one record in DrugBank providing DDIs for training the model, DeepDDI is essentially based on exploiting DDI knowledge about similar drugs. This concept also allowed us to derive previously unknown DDIs for the BTKis, which were considered in this study as a possible new class of drugs for MS [16]. The predicted DDIs of type 27 involving BTKis may be explained by DrugBank data on other BTKis for which an increased bleeding risk has been described in the literature (e.g., ibrutinib) [65]. While this approach implies that false positive findings cannot be avoided, our study offers valuable insights into potential DDIs that deserve attention and can guide actions for optimal patient care, such as the regular monitoring of peripheral blood counts to detect early signs of possible treatment-related complications.

We obtained clues for a better understanding of DDI mechanisms based on the model’s ability to predict more than one DDI type for a given drug pair. Several of the additional DDIs pointed to pharmacokinetic changes at the level of drug metabolism (DDI types 6 and 7), which may lead to adverse effects (DDI type 26). For instance, ondansetron (an antiemetic agent) was predicted to cause a decreased metabolism of zopiclone (a hypnotic agent). Zopiclone and ondansetron are both substrates of CYP3A4 and CYP2C9 [45,66,67], two of the most abundant CYP enzymes in the human liver [68]. CYP3A4 and CYP2C9 were also found to be weakly inhibited by the BTKi remibrutinib [69]. The latter finding may fit to the output, according to which a DDI of type 6 with zopiclone and/or ondansetron resulted for all five BTKis studied. As another example, the DMD teriflunomide is a weak inducer of CYP1A2 [70], a major CYP contributing to the transformation of carbamazepine (an anticonvulsant) into 2-hydroxycarbamazepine [71]. It thus seems plausible that DeepDDI predicted a decreased serum concentration of carbamazepine when combined with teriflunomide (DDI type 9). The clinical significance of this DDI remains to be established. However, it is recommended that the efficacy of drugs metabolized by CYP1A2 be monitored in patients taking teriflunomide. Advances in the understanding of DDIs, as well as their validation and proper management, are paramount to improve overall patient outcomes. The pharmacokinetic and pharmacodynamic interactions proposed in this study as potential causal mechanisms for adverse drug effects thus merit closer examination in further studies.

When there is concern of a critical DDI under the current medication or with an additional drug that needs to be prescribed, an intervention in the medication schedule may be prudent. We have shown that the predictions can be useful in suggesting alternative drugs that may avoid negative health effects while exhibiting similar pharmacological actions. For example, the therapy of trigeminal neuralgia as a symptom of MS relies on sodium channel blockers such as carbamazepine [72]. However, its use can be associated with adverse effects such as drowsiness, ataxia and hepatic abnormalities [72,73]. An overdose of acetaminophen can also induce hepatotoxicity [74]. The concurrent use of these drugs may thus increase hepatotoxic activities, as predicted (DDI type 20). Topiramate and chloroprocaine were suggested as alternative drugs as they did not yield a critical DDI with acetaminophen and share protein targets with carbamazepine. Otherwise, a different analgesic drug might be taken instead of acetaminophen. However, no further information was considered for these suggestions (e.g., drug dose, indication, route of administration or drug-target binding affinities). More specifically, topiramate and local anaesthetics have been used by some experts for the treatment of trigeminal neuralgia, but the evidence for prolonged pain relief is scarce due to the lack of well-conducted clinical trials [75,76]. Similarly, drospirenone was proposed as a substitute for spironolactone, for which DeepDDI identified an increased risk of developing hyperkalemia when combined with ramipril (DDI type 29) in agreement with the literature [77]. No significant association was found between drospirenone use and hyperkalemia [78], but drospirenone acts as a contraceptive and has antiandrogenic effects [45], which means that it may be rather an option for postmenopausal women with hypertension [79]. Therefore, the proposed replacement of drugs is often not straightforward to implement, and it remains uncertain whether greater patient safety can be achieved or not. Collaboration among healthcare professionals from different specialties and consultation with pharmacists remain vital for tailoring treatment plans to individual patient needs while avoiding preventable DDIs.

Another application of the model was the prediction of DFIs based on the chemical structures of drug and food compounds. Adverse drug reactions due to DFIs are usually avoidable by changing the diet or optimizing the timing between meals and drug intake. The results of our analysis may therefore be useful in making recommendations to MS patients about which foods and beverages should not be consumed when taking certain drugs. For example, it is well known that the concomitant use of alcohol and medications can lead to pharmacokinetic and pharmacodynamic interactions [80]. Ethanol appeared as the subject in 117 predicted DFIs and was frequently associated with increased CNS depressant activities (DDI type 54, with *n* = 20 drugs) and increased hypotensive activities (DDI type 67, with *n* = 34 drugs). Accordingly, patients undergoing drug treatment should be advised to refrain from consuming alcoholic beverages. We also obtained 6860 DFIs that potentially lead to altered in vivo concentrations of drugs, including S1P receptor modulators and BTKis, and thus may affect therapeutic outcomes in patients with MS. Previous studies have shown that the administration of BTKis with food results in higher drug exposure [81,82]. Our findings nominate individual food compounds that may increase the level of BTKis, such as thymol methyl ether via a decreased drug metabolism. Further studies may examine whether it will be useful to routinely monitor the concentration of BTKis in patient plasma, e.g., by mass spectrometry [83]. However, it should be noted that compared to DDIs, it is much more challenging to screen for DFIs because foods are composed of a wide range of chemical compounds with varying concentrations, and people’s dietary habits are very different. Moreover, a well-annotated DFI dataset remains to be established [84]. As a consequence, healthcare professionals often feel inadequately trained in counseling patients on the interactions of specific foods with drugs and the timing of food intake relative to the use of medications [85].

Several limitations of the study, apart from those already mentioned, warrant further investigation. First, our analyses focused on a limited number of drugs. The updated model, however, could also be used to predict DDIs/DFIs for other small molecule drugs, including new drugs in development for MS [17] and other diseases. We did not document the exact time intervals between the administration of different drugs and the duration of therapy, which can influence the likelihood that a DDI will actually occur. For instance, in patients receiving beta blockers and/or calcium channel blockers, an increased risk of bradycardia is especially of concern during the first two days of treatment with fingolimod [86]. We also did not ask the patients about their diet and therefore could not make any statement about the prevalence of potential DFIs in patients with MS. Moreover, the clinical consequences of previously unknown DDIs and DFIs that were predicted by the model obviously remain to be verified. While the DNN model was trained on a large number of DDIs, the extent to which factors such as the drug dosing regimen shape the risk of a DDI could not be taken into account as there is currently no information on that in DrugBank [45]. The severity level of DDIs is not specified in the database, and it is usually unclear whether the drug itself or its metabolites trigger a particular pharmacodynamic DDI. This can render their interpretation difficult. The gold standard DDI dataset was also uncertain and imbalanced and did not include drug pairs for which no DDIs have been described, which possibly impaired the classification performance. Furthermore, DeepDDI was developed to distinguish 86 DDI types [41], while in the meantime more types can be found in DrugBank [45]. Another major limitation is that the model relies on SMILES strings as input and therefore cannot be used for protein-based drugs. It is also limited to pairs of molecules, thus leaving out other types of drug interactions, such as drug-gene and multi-factorial interactions [87,88]. Perhaps another method may be even more suitable than DeepDDI for predicting DDIs in MS (Box 1). More complex DNN concepts, possibly linked to modern language models, are needed to exploit additional information (e.g., on drug targets and drug-metabolizing enzymes) to predict DDIs more accurately [36,37,38]. A future challenge will be to integrate such novel computational tools for the assessment of DDIs in the clinical setting so that physicians can swiftly identify potential risks when prescribing multiple medications and manage them effectively.

To conclude, we have updated the DeepDDI model to predict with high accuracy DDIs and DFIs for prescription and OTC drugs used by patients with MS. The analyses enabled us to identify pharmacokinetic and pharmacodynamic mechanisms that may explain adverse drug effects, to suggest alternative drugs for avoiding interacting drug combinations and to derive dietary recommendations for patients taking certain drugs. Our findings underscore the utility of a deep learning-based method for systematically screening patients’ medication plans for potential DDIs. The model might be extended to leverage additional drug features beyond structural similarities [43,89]. The integration of predictive models into clinical decision support systems may facilitate regular medication reviews and the identification of potential DDIs in clinical routine. Advances in biomedical knowledge and artificial intelligence will promote further research on drug interactions and thereby contribute to safer and more effective medication use.

## Figures and Tables

**Table 1:** Characteristics of the patient cohort (*n* = 627).

Characteristic	Statistics
**Sex, *n* (%)**	
Women	441 (70.3)
Men	186 (29.7)
**Age (years), mean ± SD**	48.6 ± 13.3
**Disease duration (years), median (range)**	10 (0–52)
**Disease course, *n* (%)**	
CIS	27 (4.3)
RRMS	388 (61.9)
SPMS	154 (24.6)
PPMS	58 (9.3)
**EDSS score, mean ± SD**	3.6 ± 2.1
**Comorbidity, *n* (%)**	
Yes	443 (70.7)
No	184 (29.3)
**Number of medications taken, mean ± SD**	
Total	5.3 ± 3.3
Rx	4.2 ± 3.0
OTC	1.1 ± 1.3
**DMD use, *n* (%)**	
Yes	389 (62.0)
No	238 (38.0)

CIS = clinically isolated syndrome, DMD = disease-modifying drug, EDSS = Expanded Disability Status Scale, OTC = over-the-counter, PPMS = primary progressive multiple sclerosis, RRMS = relapsing-remitting multiple sclerosis, Rx = prescription, SD = standard deviation, SPMS = secondary progressive multiple sclerosis.

**Table 2:** Established and investigational drugs for MS in alphabetical order.

Drug	Type	DrugBank Accession	DDIs in DrugBank ^1^
**DMDs**			
Alemtuzumab	monoclonal antibody	DB00087	821
Azathioprine	small molecule	DB00993	1276
Cladribine	small molecule	DB00242	707
Dimethyl fumarate	small molecule	DB08908	340
Diroximel fumarate	small molecule	DB14783	342
Fingolimod	small molecule	DB08868	560
Glatiramer acetate	peptides	DB05259	340
Interferon beta-1a	recombinant protein	DB00060	203
Interferon beta-1b	recombinant protein	DB00068	643
Mitoxantrone	small molecule	DB01204	653
Monomethyl fumarate	small molecule	DB14219	340
Natalizumab	monoclonal antibody	DB00108	651
Ocrelizumab	monoclonal antibody	DB11988	650
Ofatumumab	monoclonal antibody	DB06650	685
Ozanimod	small molecule	DB12612	589
Peginterferon beta-1a	recombinant protein	DB09122	544
Ponesimod	small molecule	DB12016	965
Siponimod	small molecule	DB12371	1318
Teriflunomide	small molecule	DB08880	782
Ublituximab	monoclonal antibody	DB11850	360
**BTKis**			
Evobrutinib	small molecule	DB15170	0
Fenebrutinib	small molecule	DB14785	0
Orelabrutinib	small molecule	DB16272	0
Remibrutinib	small molecule	DB16852	0
Tolebrutinib	small molecule	–	–

^1^ release version 5.1.10. BTKi = Bruton’s tyrosine kinase inhibitor under investigation in MS, DDI = drug-drug interaction, DMD = disease-modifying drug approved for the treatment of MS, MS = multiple sclerosis.

## Data Availability

The data supporting the findings of this study are available in the article and/or Supplementary Materials. The updated DeepDDI model is available from https://zenodo.org/record/8341065 (published on 13 September 2023).

## Supplementary Materials

The following supporting information can be downloaded at: https://www.mdpi.com/article/10.3390/pharmaceutics16010003/s1, Figure S1: Chemical structures of DMDs for MS as well as BTKis; Figure S2: Evaluation metrics for the DeepDDI model update; Figure S3: Prediction accuracy of the updated DeepDDI model for each DDI type; Figure S4: Frequency of DDIs with current and emerging drugs for MS. Supplemental Table S1 (XLSX): General sentence structures of the 86 DDI types that are distinguished by DeepDDI. Supplemental Table S2 (XLSX): Drug compounds that were used by the patients with MS. Supplemental Table S3 (XLSX): DDIs determined with DeepDDI between drug compounds taken by the patients with MS as well as DMDs and BTKis. Supplemental Table S4 (XLSX): DFIs determined with DeepDDI for drug compounds taken by the patients with MS as well as DMDs and BTKis.

## Abbreviations

BTK = Bruton’s tyrosine kinase, BTKi = Bruton’s tyrosine kinase inhibitor, CIS = clinically isolated syndrome, CNS = central nervous system, CYP = cytochrome P450, DDI = drug-drug interaction, DFI = drug-food interaction, DMD = disease-modifying drug, DNN = deep neural network, EDSS = Expanded Disability Status Scale, MS = multiple sclerosis, OTC = over-the-counter, PCA = principal component analysis, PPMS = primary progressive multiple sclerosis, RRMS = relapsing-remitting multiple sclerosis, Rx = prescription, S1P = sphingosine-1-phosphate, SD = standard deviation, SMILES = simplified molecular-input line-entry system, SPMS = secondary progressive multiple sclerosis.

## References

[1] Filippi M., Bar-Or A., Piehl F., Preziosa P., Solari A., Vukusic S., Rocca M.A. (2018). Multiple sclerosis. Nat. Rev. Dis. Primers.

[2] Walton C., King R., Rechtman L., Kaye W., Leray E., Marrie R.A., Robertson N., La Rocca N., Uitdehaag B., van der Mei I. (2020). Rising prevalence of multiple sclerosis worldwide: Insights from the Atlas of MS, third edition. Mult. Scler..

[3] Rommer P.S., Eichstädt K., Ellenberger D., Flachenecker P., Friede T., Haas J., Kleinschnitz C., Pöhlau D., Rienhoff O., Stahmann A. (2019). Symptomatology and symptomatic treatment in multiple sclerosis: Results from a nationwide MS registry. Mult. Scler..

[4] Lublin F.D., Reingold S.C., Cohen J.A., Cutter G.R., Sørensen P.S., Thompson A.J., Wolinsky J.S., Balcer L.J., Banwell B., Barkhof F. (2014). Defining the clinical course of multiple sclerosis: The 2013 revisions. Neurology.

[5] Thompson A.J., Banwell B.L., Barkhof F., Carroll W.M., Coetzee T., Comi G., Correale J., Fazekas F., Filippi M., Freedman M.S. (2018). Diagnosis of multiple sclerosis: 2017 revisions of the McDonald criteria. Lancet Neurol..

[6] Goodin D.S. (2016). The epidemiology of multiple sclerosis: Insights to a causal cascade. Handb. Clin. Neurol..

[7] McGinley M.P., Goldschmidt C.H., Rae-Grant A.D. (2021). Diagnosis and Treatment of Multiple Sclerosis: A Review. JAMA.

[8] Hauser S.L., Cree B.A.C. (2020). Treatment of Multiple Sclerosis: A Review. Am. J. Med..

[9] Yang J.H., Rempe T., Whitmire N., Dunn-Pirio A., Graves J.S. (2022). Therapeutic Advances in Multiple Sclerosis. Front. Neurol..

[10] Wiendl H., Gold R., Berger T., Derfuss T., Linker R., Mäurer M., Aktas O., Baum K., Berghoff M., Bittner S. (2021). Multiple Sclerosis Therapy Consensus Group (MSTCG): Position statement on disease-modifying therapies for multiple sclerosis (white paper). Ther. Adv. Neurol. Disord..

[11] Rommer P.S., Milo R., Han M.H., Satyanarayan S., Sellner J., Hauer L., Illes Z., Warnke C., Laurent S., Weber M.S. (2019). Immunological Aspects of Approved MS Therapeutics. Front. Immunol..

[12] Moiola L., Rommer P.S., Zettl U.K. (2020). Prevention and management of adverse effects of disease modifying treatments in multiple sclerosis. Curr. Opin. Neurol..

[13] Rommer P.S., Zettl U.K. (2018). Managing the side effects of multiple sclerosis therapy: Pharmacotherapy options for patients. Expert. Opin. Pharmacother..

[14] McDonald C., Xanthopoulos C., Kostareli E. (2021). The role of Bruton’s tyrosine kinase in the immune system and disease. Immunology.

[15] Carnero Contentti E., Correale J. (2022). Current Perspectives: Evidence to Date on BTK Inhibitors in the Management of Multiple Sclerosis. Drug Des. Dev. Ther..

[16] Krämer J., Bar-Or A., Turner T.J., Wiendl H. (2023). Bruton tyrosine kinase inhibitors for multiple sclerosis. Nat. Rev. Neurol..

[17] Cree B.A.C., Hartung H.P., Barnett M. (2022). New drugs for multiple sclerosis: New treatment algorithms. Curr. Opin. Neurol..

[18] Kochs L., Wegener S., Sühnel A., Voigt K., Zettl U.K. (2014). The use of complementary and alternative medicine in patients with multiple sclerosis: A longitudinal study. Complement. Ther. Med..

[19] Rommer P.S., König N., Sühnel A., Zettl U.K. (2018). Coping behavior in multiple sclerosis-complementary and alternative medicine: A cross-sectional study. CNS Neurosci. Ther..

[20] Frahm N., Hecker M., Zettl U.K. (2021). Polypharmacy in Chronic Neurological Diseases: Multiple Sclerosis, Dementia and Parkinson’s Disease. Curr. Pharm. Des..

[21] Hecker M., Frahm N., Bachmann P., Debus J.L., Haker M.C., Mashhadiakbar P., Langhorst S.E., Baldt J., Streckenbach B., Heidler F. (2022). Screening for severe drug-drug interactions in patients with multiple sclerosis: A comparison of three drug interaction databases. Front. Pharmacol..

[22] Hakkola J., Hukkanen J., Turpeinen M., Pelkonen O. (2020). Inhibition and induction of CYP enzymes in humans: An update. Arch. Toxicol..

[23] Niu J., Straubinger R.M., Mager D.E. (2019). Pharmacodynamic Drug-Drug Interactions. Clin. Pharmacol. Ther..

[24] Spanakis M., Patelarou E., Patelarou A. (2022). Drug-Food Interactions with a Focus on Mediterranean Diet. Appl. Sci..

[25] Jalusic K.O., Ellenberger D., Rommer P., Stahmann A., Zettl U., Berger K. (2021). Effect of applying inclusion and exclusion criteria of phase III clinical trials to multiple sclerosis patients in routine clinical care. Mult. Scler..

[26] Zitnik M., Nguyen F., Wang B., Leskovec J., Goldenberg A., Hoffman M.M. (2019). Machine Learning for Integrating Data in Biology and Medicine: Principles, Practice, and Opportunities. Inf. Fusion.

[27] Lu C., Di L. (2020). In vitro and in vivo methods to assess pharmacokinetic drug-drug interactions in drug discovery and development. Biopharm. Drug Dispos..

[28] Vinarov Z., Butler J., Kesisoglou F., Koziolek M., Augustijns P. (2023). Assessment of food effects during clinical development. Int. J. Pharm..

[29] Roblek T., Vaupotic T., Mrhar A., Lainscak M. (2015). Drug-drug interaction software in clinical practice: A systematic review. Eur. J. Clin. Pharmacol..

[30] Marcath L.A., Xi J., Hoylman E.K., Kidwell K.M., Kraft S.L., Hertz D.L. (2018). Comparison of Nine Tools for Screening Drug-Drug Interactions of Oral Oncolytics. J. Oncol. Pract..

[31] Hines L.E., Malone D.C., Murphy J.E. (2012). Recommendations for generating, evaluating, and implementing drug-drug interaction evidence. Pharmacotherapy.

[32] Kheshti R., Aalipour M., Namazi S. (2016). A comparison of five common drug-drug interaction software programs regarding accuracy and comprehensiveness. J. Res. Pharm. Pract..

[33] Sancar M., Kaşik A., Okuyan B., Batuhan S., Izzettin F.V. (2019). Determination of Potential Drug-Drug Interactions Using Various Software Programs in a Community Pharmacy Setting. Turk. J. Pharm. Sci..

[34] Armahizer M.J., Kane-Gill S.L., Smithburger P.L., Anthes A.M., Seybert A.L. (2013). Comparing drug-drug interaction severity ratings between bedside clinicians and proprietary databases. Int. Sch. Res. Not..

[35] Zheng W.Y., Richardson L.C., Li L., Day R.O., Westbrook J.I., Baysari M.T. (2018). Drug-drug interactions and their harmful effects in hospitalised patients: A systematic review and meta-analysis. Eur. J. Clin. Pharmacol..

[36] Han K., Cao P., Wang Y., Xie F., Ma J., Yu M., Wang J., Xu Y., Zhang Y., Wan J. (2022). A Review of Approaches for Predicting Drug-Drug Interactions Based on Machine Learning. Front. Pharmacol..

[37] Hong E., Jeon J., Kim H.U. (2023). Recent development of machine learning models for the prediction of drug-drug interactions. Korean J. Chem. Eng..

[38] Lin X., Dai L., Zhou Y., Yu Z.G., Zhang W., Shi J.Y., Cao D.S., Zeng L., Chen H., Song B. (2023). Comprehensive evaluation of deep and graph learning on drug-drug interactions prediction. Brief. Bioinform..

[39] Percha B., Altman R.B. (2013). Informatics confronts drug-drug interactions. Trends Pharmacol. Sci..

[40] Cheng F., Zhao Z. (2014). Machine learning-based prediction of drug-drug interactions by integrating drug phenotypic, therapeutic, chemical, and genomic properties. J. Am. Med. Inform. Assoc..

[41] Ryu J.Y., Kim H.U., Lee S.Y. (2018). Deep learning improves prediction of drug-drug and drug-food interactions. Proc. Natl. Acad. Sci. USA.

[42] Zitnik M., Agrawal M., Leskovec J. (2018). Modeling polypharmacy side effects with graph convolutional networks. Bioinformatics.

[43] Deng Y., Xu X., Qiu Y., Xia J., Zhang W., Liu S. (2020). A multimodal deep learning framework for predicting drug-drug interaction events. Bioinformatics.

[44] Udrescu M., Ardelean S.M., Udrescu L. (2022). The curse and blessing of abundance-the evolution of drug interaction databases and their impact on drug network analysis. Gigascience.

[45] Wishart D.S., Feunang Y.D., Guo A.C., Lo E.J., Marcu A., Grant J.R., Sajed T., Johnson D., Li C., Sayeeda Z. (2018). DrugBank 5.0: A major update to the DrugBank database for 2018. Nucleic Acids Res..

[46] Debus J.L., Bachmann P., Frahm N., Mashhadiakbar P., Langhorst S.E., Streckenbach B., Baldt J., Heidler F., Hecker M., Zettl U.K. (2022). Associated factors of potential drug-drug interactions and drug-food interactions in patients with multiple sclerosis. Ther. Adv. Chronic Dis..

[47] Bachmann P., Frahm N., Debus J.L., Mashhadiakbar P., Langhorst S.E., Streckenbach B., Baldt J., Heidler F., Hecker M., Zettl U.K. (2022). Prevalence and Severity of Potential Drug-Drug Interactions in Patients with Multiple Sclerosis with and without Polypharmacy. Pharmaceutics.

[48] Kurtzke J.F. (1983). Rating neurologic impairment in multiple sclerosis: An expanded disability status scale (EDSS). Neurology.

[49] Amin M., Hersh C.M. (2023). Updates and advances in multiple sclerosis neurotherapeutics. Neurodegener. Dis. Manag..

[50] Lee A. (2023). Ublituximab: First Approval. Drugs.

[51] Kim S., Chen J., Cheng T., Gindulyte A., He J., He S., Li Q., Shoemaker B.A., Thiessen P.A., Yu B. (2023). PubChem 2023 update. Nucleic Acids Res..

[52] Cereto-Massagué A., Ojeda M.J., Valls C., Mulero M., Garcia-Vallvé S., Pujadas G. (2015). Molecular fingerprint similarity search in virtual screening. Methods.

[53] Kingma D.P., Ba J. (2017). Adam: A method for stochastic optimization. arXiv.

[54] Abadi M., Agarwal A., Barham P., Brevdo E., Chen Z., Citro C., Corrado G.S., Davis A., Dean J., Devin M. (2016). TensorFlow: Large-Scale Machine Learning on Heterogeneous Distributed Systems. arXiv.

[55] Shannon P., Markiel A., Ozier O., Baliga N.S., Wang J.T., Ramage D., Amin N., Schwikowski B., Ideker T. (2003). Cytoscape: A software environment for integrated models of biomolecular interaction networks. Genome Res..

[56] Frahm N., Hecker M., Langhorst S.E., Mashhadiakbar P., Haker M.C., Zettl U.K. (2020). The risk of polypharmacy, comorbidities and drug-drug interactions in women of childbearing age with multiple sclerosis. Ther. Adv. Neurol. Disord..

[57] Moore N., Pollack C., Butkerait P. (2015). Adverse drug reactions and drug-drug interactions with over-the-counter NSAIDs. Ther. Clin. Risk Manag..

[58] Ahmed S., Zhou Z., Zhou J., Chen S.Q. (2016). Pharmacogenomics of Drug Metabolizing Enzymes and Transporters: Relevance to Precision Medicine. Genom. Proteom. Bioinform..

[59] Holstiege J., Akmatov M.K., Klimke K., Dammertz L., Kohring C., Marx C., Frahm N., Peters M., Ellenberger D., Zettl U.K. (2022). Trends in administrative prevalence of multiple sclerosis and utilization patterns of disease modifying drugs in Germany. Mult. Scler. Relat. Disord..

[60] Wundes A., Kraft G.H., Bowen J.D., Gooley T.A., Nash R.A. (2010). Mitoxantrone for worsening multiple sclerosis: Tolerability, toxicity, adherence and efficacy in the clinical setting. Clin. Neurol. Neurosurg..

[61] Espinola-Klein C. (2022). When and How to Combine Antiplatelet and Anticoagulant Drugs?. Hamostaseologie.

[62] Giovannoni G., Mathews J. (2022). Cladribine Tablets for Relapsing-Remitting Multiple Sclerosis: A Clinician’s Review. Neurol. Ther..

[63] Zhou A., Han Q., Song H., Zi J., Ma J., Ge Z. (2019). Efficacy and toxicity of cladribine for the treatment of refractory acute myeloid leukemia: A meta-analysis. Drug Des. Dev. Ther..

[64] Pagano L., Criscuolo M., Broccoli A., Piciocchi A., Varettoni M., Galli E., Anastasia A., Cantonetti M., Trentin L., Kovalchuk S. (2022). Long-term follow-up of cladribine treatment in hairy cell leukemia: 30-year experience in a multicentric Italian study. Blood Cancer J..

[65] von Hundelshausen P., Siess W. (2021). Bleeding by Bruton Tyrosine Kinase-Inhibitors: Dependency on Drug Type and Disease. Cancers.

[66] Zhou S.F., Zhou Z.W., Yang L.P., Cai J.P. (2009). Substrates, inducers, inhibitors and structure-activity relationships of human Cytochrome P450 2C9 and implications in drug development. Curr. Med. Chem..

[67] Rendic S. (2002). Summary of information on human CYP enzymes: Human P450 metabolism data. Drug Metab. Rev..

[68] Zanger U.M., Schwab M. (2013). Cytochrome P450 enzymes in drug metabolism: Regulation of gene expression, enzyme activities, and impact of genetic variation. Pharmacol. Ther..

[69] Schiller H., Huth F., Schuhler C., Drollmann A., Kaul M., Woessner R., Shah B., Weis W., End P. (2022). Novel Bruton’s tyrosine kinase inhibitor remibrutinib: Assessment of drug-drug interaction potential as a perpetrator of cytochrome P450 enzymes and drug transporters and the impact of covalent binding on possible drug interactions. Eur. J. Pharm. Sci..

[70] Miller A.E. (2021). An updated review of teriflunomide’s use in multiple sclerosis. Neurodegener. Dis. Manag..

[71] Wang B., Zhou S.F. (2009). Synthetic and natural compounds that interact with human cytochrome P450 1A2 and implications in drug development. Curr. Med. Chem..

[72] Di Stefano G., Maarbjerg S., Truini A. (2019). Trigeminal neuralgia secondary to multiple sclerosis: From the clinical picture to the treatment options. J. Headache Pain.

[73] Kalapos M.P. (2002). Carbamazepine-provoked hepatotoxicity and possible aetiopathological role of glutathione in the events. Retrospective review of old data and call for new investigation. Advers. Drug React. Toxicol. Rev..

[74] Yoon E., Babar A., Choudhary M., Kutner M., Pyrsopoulos N. (2016). Acetaminophen-Induced Hepatotoxicity: A Comprehensive Update. J. Clin. Transl. Hepatol..

[75] Domingues R.B., Kuster G.W., Aquino C.C. (2007). Treatment of trigeminal neuralgia with low doses of topiramate. Arq. Neuropsiquiatr..

[76] Bendtsen L., Zakrzewska J.M., Heinskou T.B., Hodaie M., Leal P.R.L., Nurmikko T., Obermann M., Cruccu G., Maarbjerg S. (2020). Advances in diagnosis, classification, pathophysiology, and management of trigeminal neuralgia. Lancet Neurol..

[77] Raebel M.A. (2012). Hyperkalemia associated with use of angiotensin-converting enzyme inhibitors and angiotensin receptor blockers. Cardiovasc. Ther..

[78] Bird S.T., Pepe S.R., Etminan M., Liu X., Brophy J.M., Delaney J.A. (2011). The association between drospirenone and hyperkalemia: A comparative-safety study. BMC Clin. Pharmacol..

[79] Mallareddy M., Hanes V., White W.B. (2007). Drospirenone, a new progestogen, for postmenopausal women with hypertension. Drugs Aging.

[80] Johnson B.A., Seneviratne C. (2014). Alcohol-medical drug interactions. Handb. Clin. Neurol..

[81] Papasouliotis O., Mitchell D., Girard P., Dangond F., Dyroff M. (2022). Determination of a clinically effective evobrutinib dose: Exposure-response analyses of a phase II relapsing multiple sclerosis study. Clin. Transl. Sci..

[82] Reich D.S., Arnold D.L., Vermersch P., Bar-Or A., Fox R.J., Matta A., Turner T., Wallström E., Zhang X., Mareš M. (2021). Tolebrutinib Phase 2b Study Group. Safety and efficacy of tolebrutinib, an oral brain-penetrant BTK inhibitor, in relapsing multiple sclerosis: A phase 2b, randomised, double-blind, placebo-controlled trial. Lancet Neurol..

[83] Ji S., Liu X., Ha J., Ai L., Li Z. (2023). Quantification of orelabrutinib in human plasma and cerebrospinal fluid by liquid chromatography tandem mass spectrometry. J. Chromatogr. B Analyt Technol. Biomed. Life Sci..

[84] Kim S., Choi Y., Won J.H., Mi Oh J., Lee H. (2022). An annotated corpus from biomedical articles to construct a drug-food interaction database. J. Biomed. Inform..

[85] Osuala E.C., Ojewole E.B. (2021). Knowledge, attitudes and practices of healthcare professionals regarding drug-food interactions: A scoping review. Int. J. Pharm. Pract..

[86] Gold R., Comi G., Palace J., Siever A., Gottschalk R., Bijarnia M., von Rosenstiel P., Tomic D., Kappos L., FIRST Study Investigators (2014). Assessment of cardiac safety during fingolimod treatment initiation in a real-world relapsing multiple sclerosis population: A phase 3b, open-label study. J. Neurol..

[87] Türk D., Fuhr L.M., Marok F.Z., Rüdesheim S., Kühn A., Selzer D., Schwab M., Lehr T. (2021). Novel models for the prediction of drug-gene interactions. Expert. Opin. Drug Metab. Toxicol..

[88] Bechtold B., Clarke J. (2021). Multi-factorial pharmacokinetic interactions: Unraveling complexities in precision drug therapy. Expert. Opin. Drug Metab. Toxicol..

[89] Lee G., Park C., Ahn J. (2019). Novel deep learning model for more accurate prediction of drug-drug interaction effects. BMC Bioinform..

