# Supplementary material for: Update and Application of a Deep Learning Model for the Prediction of Interactions between Drugs Used by Patients with Multiple Sclerosis
Source: Pharmaceutics. 2023 Dec 19;16(1):3. doi: 10.3390/pharmaceutics16010003 (PMC10819178; doi:10.3390/pharmaceutics16010003)
Supplement: Supplementary file 1 [file pharmaceutics-16-00003-s001.zip › Supplemental_Document.pdf]

Supplemental Document for

Update and application of a deep learning model for the prediction of interactions between drugs used by patients with multiple sclerosis

Michael Hecker[1,*], Niklas Frahm[1], Uwe Klaus Zettl[1]

[1] Rostock University Medical Center, Department of Neurology, Division of Neuroimmunology, Gehlsheimer Str. 20, 18147 Rostock, Germany

[*] corresponding author: Michael Hecker, e-mail: michael.hecker@rocketmail.com,
phone: +49 381 494-5890,
ORCID ID: 0000-0001-7015-3094

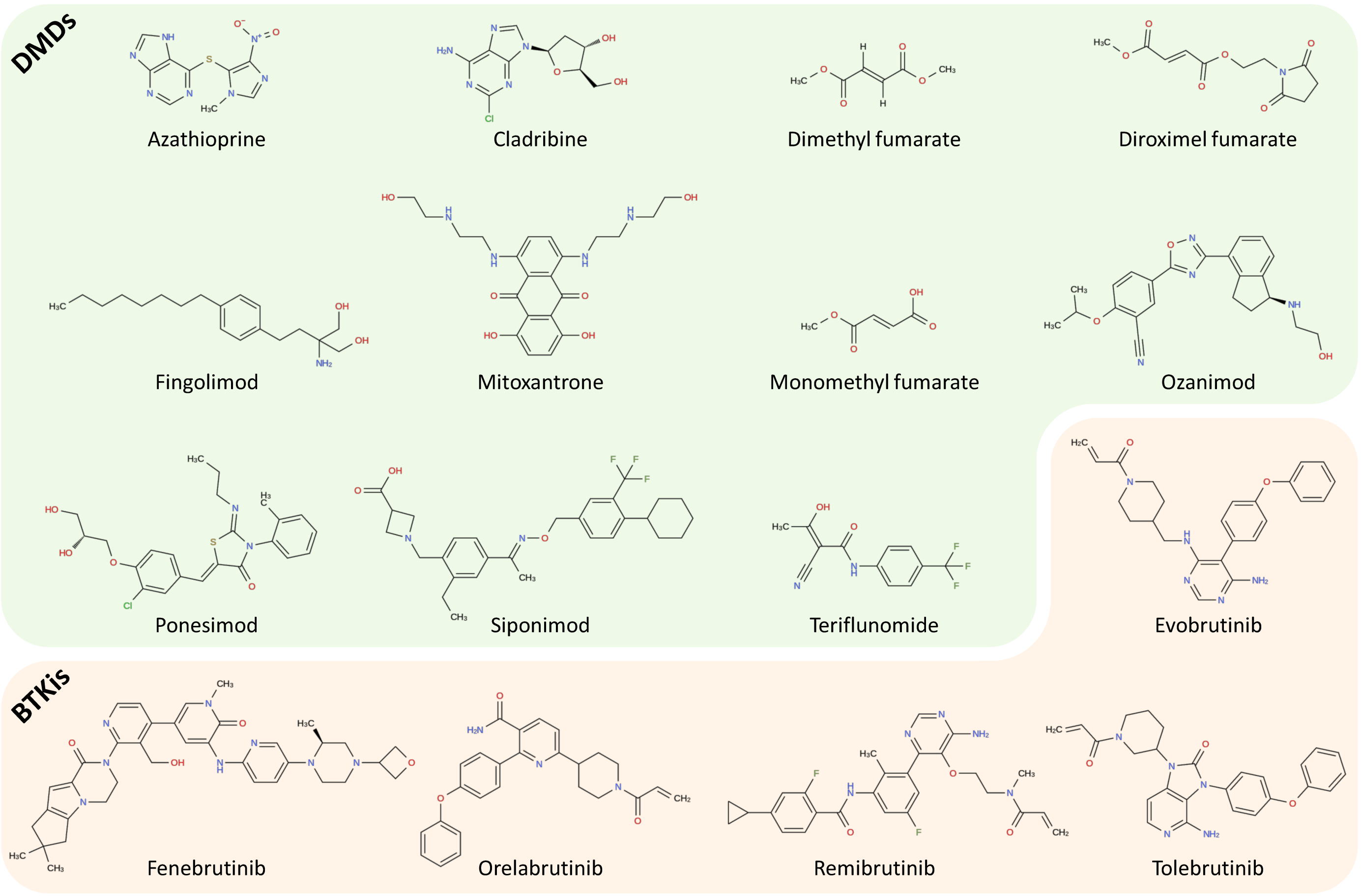

Supplemental Figure S1 : Chemical structures of DMDs for MS as well as BTKis.

The two-dimensional structures were visualized with the Indigo toolkit (version 1.10.0) for Python (version 3.6.15) using the drugs' canonical SMILES representations. Protein- or peptide-based DMDs for the treatment of MS cannot be used as input for DeepDDI and are thus not shown here.

BTKi = Bruton's tyrosine kinase inhibitor being tested in clinical trials in MS, DMD = disease-modifying drug, MS = multiple sclerosis, SMILES = simplified molecular-input line-entry system

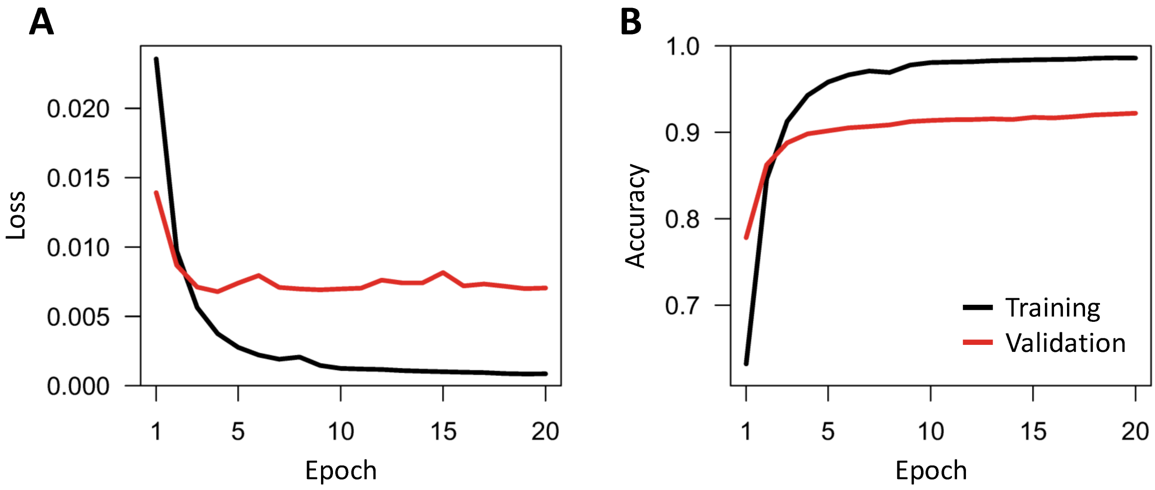

Supplemental Figure S2 : Evaluation metrics for the DeepDDI model update.

The original (pre-trained) model was trained for another 20 epochs using the new gold standard (*n*=1046705 DDIs) from the DrugBank database release 5.1.10. This was done with the Python library TensorFlow (version 2.6.4) on a GPU P100 provided by Kaggle (execution time: ~30 seconds per epoch). **A**) Decreasing mean loss curves and **B**) increasing categorical accuracy curves show the model fitting (i.e., the optimization of the ~34 million trainable parameters of the DNN). The final updated model achieved accuracies of 99.04%, 92.21% and 92.14% on the training set, validation set and testing set, respectively.

DNN = deep neural network, GPU = graphics processing unit

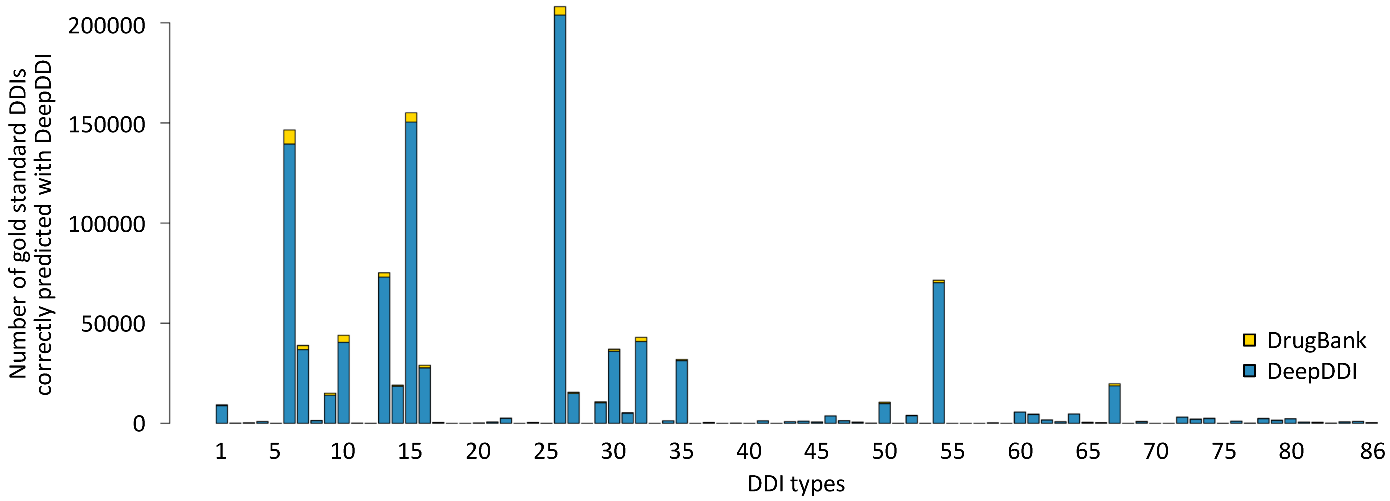

Supplemental Figure S3 : Prediction accuracy of the updated DeepDDI model for each DDI type.

From the DrugBank database (release 5.1.10), we determined 1046705 DDIs that belong to the 86 DDI types distinguished by DeepDDI. These DDIs connect 3423 different small molecule drugs. Based on the chemical structures of the drugs (in SMILES), 1009050 of the DDIs (96.40%) were correctly predicted by DeepDDI after fitting the model to the new gold standard.

DDI = drug-drug interaction, SMILES = simplified molecular-input line-entry system

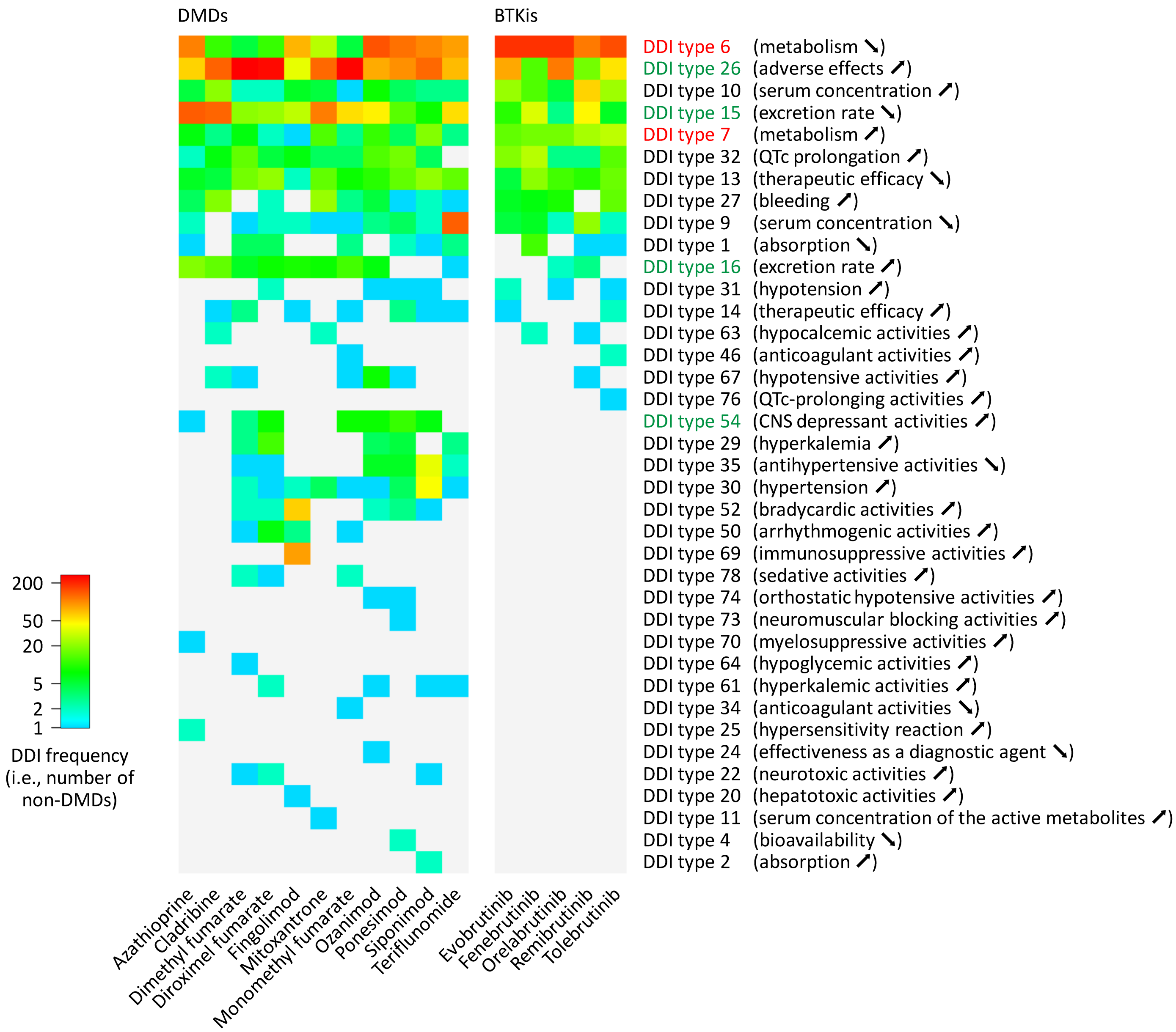

Supplemental Figure S4 : Frequency of DDIs with current and emerging drugs for MS.

A total of 306 different non-DMDs were used by the patients with MS (N=627). This heatmap shows how many of these drugs were predicted by DeepDDI to cause a DDI of a particular type when used in combination with a DMD or BTKi. Please note that this analysis was limited to small molecule drugs with well-defined chemical structure. DDIs of 38 different types were predicted for the DMDs and BTKis. The remaining 48 DDI types are not shown here. The rows of the matrix are sorted by DDI frequency for BTKis. Large values are shown in red, as depicted in the rainbow color map on the left. DDI types written in red and green indicate that, on average, a higher and lower number of drugs, respectively, were found to have such DDIs with BTKis than with DMDs (Welch *t*-test *p*-values <0.05). Key terms from the descriptions are given next to each DDI type.

BTKi = Bruton's tyrosine kinase inhibitor under investigation in MS, CNS = central nervous system, DDI = drug-drug interaction, DMD = disease-modifying drug approved for the treatment of MS, MS = multiple sclerosis, QTc = heart rate-corrected QT interval, ⬈ = increased, ⬊ = decreased
